# Effect of course coordinator behavior and motivation on students’ achievement: Results from five curriculum blocks of two undergraduate student cohorts at King Saud bin Abdulaziz University of Health Sciences

**DOI:** 10.12669/pjms.312.7016

**Published:** 2015

**Authors:** Ibrahim Al-Alwan, Lubna Ansari Baig, Motasim Badri, Mohi Eldin Magzoub, Sarah Alyousif

**Affiliations:** 1 Prof. Ibrahim Al-Alwan, MD. Dean, College of Medicine, King Saud bin Abdulaziz University of Health Sciences (KSAU-HS), Riyadh, Kingdom of Saudi Arabia; 2 Dr. Lubna A. Baig, MBBS, MPH, FCPS, PhD. Dean and Professor, APPNA Institute of Public Health Jinnah Sindh Medical University, Karachi, Pakistan; 3 Dr. Motasim Badri, PhD. Associate Professor, Department of Medical Education, King Saud bin Abdulaziz University of Health Sciences (KSAU-HS), Riyadh, Kingdom of Saudi Arabia; 4 Prof. Mohi Eldin Magzoub, MD, PhD. Professor, Chairman, Department of Medical Education, King Saud bin Abdulaziz University of Health Sciences (KSAU-HS), Riyadh, Kingdom of Saudi Arabia; 5 Dr. Sarah Alyousif, PhD. College of Pharmacy, King Saud bin Abdulaziz University of Health Sciences (KSAU-HS), Riyadh, Kingdom of Saudi Arabia

**Keywords:** Curriculum, Medical Faculty, Motivation, Evaluation, Medical Education, Medical Students

## Abstract

**Objective:**

The purpose of the study was to assess the relationship between students’ perception of course/block coordinators performance and attributes with students’ assessment scores in respective courses.

**Methods:**

This retrospective data based study was conducted at the College of Medicine, King Saud bin Abdulaziz University of Health Sciences (KSAU-HS). It was started in March 2013 and completed in June 2013 after the graduation of the fourth cohort. Exam score of 3^rd^ and 4^th^ cohort of students from the courses taught in the last two years of medical school were correlated with faculty and block evaluation done by the students. Scores from mid-block MCQs, portfolio scores, OSCEs and end-of-block MCQs were obtained.

**Results:**

The Mean scores of all the assessments for all five blocks were not significantly different for both batches. There was significant difference between block coordinators for students’ score on portfolio, midterm exam and the final written exam. The students’ Score in OSCE had significantly strong correlation with quality of station monitors, coverage of content and flow between stations. Student’s perception of the commitment and motivation of the coordinator was strongly correlated with block organization, availability of clinical cases, performance of block coordinator, cooperation with students, and organization of clinical activities.

**Conclusions:**

Block coordinator’s motivation and commitment affects quality of block organization and student`s success. Faculty training programs should include block management competencies and components identified through self-determination theory for improving the intrinsic motivation for students success.

## INTRODUCTION

The quality of an educational program is affected by multiple factors and generally measured through students’ achievement in exams (GPA or percent score) and or satisfaction with the program.^1^,^2^ Two important factors that affect the scores of the students in an educational program have been identified as the knowledge and ability of faculty supervisors in conducting the course.^3^,^4^ Other important factors include the curricular design, administrative skills of the supervisors, learning resources and environment.^5^

Lieff and colleagues reported that facilitating the growth of academic identity has the potential to increase faculty motivation, satisfaction, and productivity.^6^ Factors salient to the formation of academic identity were mushroomed under personal (cognitive and emotional factors unique to each individual); relational (connections and interactions with others); and contextual (the program itself and external work environments) domains. Therefore, faculty development initiatives should incorporate these factors when considering issues of program design, portfolio development, and implementation.^6^

Psychology literature informs that motivation is dependent on the fulfillment of three basic psychological needs which are autonomy, competence and relatedness.^7^,^8^ The principles that aid in fulfillment of these may progress from factors of extrinsic to intrinsic motivation i.e. from a motivation to intrinsic motivation.^9^ The faculty’s role in students’ achievement has however never been measured in the Saudi Arabian context. This study aimed to explore the relationship between students’ perceived course coordinators’ performance, (inclusive of commitment and motivation) and student assessment scores in multiple courses of two cohorts in the College of Medicine at King Saud bin Abdulaziz University, Riyadh. Arguments from the study also contribute towards adducing empirical evidence for the relationship of students’ perception of faculty motivation with their learning.

## METHODS

The College of Medicine in King Saud bin Abdulaziz University for Health Sciences, Riyadh, has a four year hybrid problem-based learning (PBL) program adapted from the University of Sydney. The program is delivered as organ-system based courses (referred to as blocks) in the first two years and then in discipline based courses (referred to as blocks) inclusive of clerkship in medicine, surgery, pediatrics, gynecology and obstetrics, and family medicine in the later two years. Block refers to the organ system courses in the preclinical years and discipline or specialty rotations in the clinical years. The block is managed by a faculty member referred to as the block coordinator. The responsibilities of the block coordinator include, a) organization and smooth functioning of the block, b) assigning tutors and attachments for the students, c) developing the assessment tools with other faculty members in the block, d) dealing with students’ learning issues during the block.

Block coordinators’ evaluation tool was developed by a team of multidisciplinary faculty that included medical education and program evaluation experts and block coordinators. The key variables in the block coordinators’ evaluation form included capability of block organization, rapport with the students, availability, commitment and motivation, critical thinking ability and factors that ensured smooth functioning of the block.

This study was done with the third and fourth cohort (referred to as batch at KSAU-HS) of students’ during the latter two clinical years of their study in the college. Data from the block evaluations and assessment scores of all the 32 and 38 male students of the third and fourth batch respectively were included in the study. A break-up of the scores in mid-block MCQs, Mini-CEX, OSCEs and end-of-block MCQs were obtained. The study was approved by the Research Ethics Committee and confidentiality and, compliance with institutional policies was ensured. The study was started in March 2013 and completed in June 2013 after the graduation of the fourth cohort.

### Analysis

There were 32 students in the third batch and 38 in the fourth batch who were assessed in the disciplines of: family medicine, general gynecology and obstetrics, general pediatrics, pediatric surgery, and rheumatology. Four males and two female coordinators organized the curriculum in five clinical disciplines and they were evaluated by the students through a structured questionnaire with a lickert scale. The block coordinators for all the blocks except gynecology and obstetrics were the same faculty members. In genecology and obstetrics there were different coordinators for both batches. Students also evaluated the quality of the block on a Lickert type scale (1=strongly disagree, 2= disagree, 3=neither agree nor disagree, 4=agree, 5=strongly agree).

Data was entered and analyzed on SPSS version 19. Spearman’s Correlations and ANOVA with post Hoc analysis (bonferroni correction) was used for assessing significant relationships and p-value of less than 0.05 was considered as significant. Reliability of the questionnaire was Cronbach’s alpha of 0.91.

## RESULTS

There was no effect of the coordinators age, demographics, and years of experience on students’ scores. Out of the six coordinators that organized the blocks only one had a Masters in Medical Education and the others had attended some educational workshops. One female faculty member from the Gynecology and Obstetrics blocks had a Master’s degree in medical education (referred to as Coordinator 1 in Table-I). There was a significant difference in the scores of the students with different preceptors. On Boneferoni correction we found that the students with the coordinator who had a Masters Degree had significantly higher scores in Portfolio assessment (p<0.0001), Mean midterm exam (p<0.001), and the total score (p<0.0001). (Table-I)

**Table-I:** Significant Difference in Student Outcomes among Different Coordinators (Cord).

Students Assessment	Rheumatology Mean (SD)	Pediatric surgery Mean (SD)	Family Medicine Mean (SD)	Gynecology/Obstetrics Cord^1^ Mean (SD)	Gynecology/Obstetrics Cord 2 Mean (SD)	General Pediatrics Mean (SD)	P-value
Mean Mid-term exam Out of 15	11.19 (1.19)	10.69 (0.76)	11.65 (0.44)	11.37 (0.66)	10.55 (1.92)	10.99 (0.23)	0.011
Class Mean Portfolio Out of 25	17.83 (0.46)	18.38 (0.02)	19.68 (0.42)	20.99 (1.80)	16.97 (1.25)	18.40 (0.43)	0.001
Class Mean Final written Out of 25	24.97 (0.32)	21.35 (2.67)	24.20 (0.16)	24.08 (1.47)	25.28 (2.10)	25.49 (0.98)	0.001
Class Mean OSCE Out of 30	24.97 (0.32)	21.0 (2.67)	24.20 (0.16)	24.08 (1.96)	25.28 (1.51)	25.49 (0.98)	0.000
Data Interpretation Out of 5	3.71 (0.35)	4.19 (0.21)	4.02 (0.26)	3.23 (0.70)	3.92 (0.76)	3.94 (0.41)	0.001
Class mean of total score Out of 100	78.68 (1.56)	76.13 (5.33)	80.47 (0.04)	83.49 (3.38)	79.20 (5.14)	81.73 (1.73)	0.000

1 Coordinator in Gynaecology and obstetrics with a Masters Degree in Medical Education

The average scores of all the assessments for all five blocks were not significantly different for both batches. There were significantly higher scores in the midterm exam (p<0.031) and data interpretation (p<0.002) for batch 3 students in Rheumatology. In general pediatrics batch 3 students had significantly (p<0.003) high scores in portfolio assessment. In Gynecology and obstetrics batch 4 students got a high mean score in data interpretation (p<0.016). (Table-II)

**Table II:** Comparison of Mean Scores of five rotations for Batch 3 and 4 undergraduate medical students

Discipline	Batch	Class Mean Final written Out of 25 Mean (SD)	Class Mean Mid-term Out of 15 Mean (SD)	Class Mean Portfolio Out of 25 Mean (SD)	Class Mean OSCE Out of 30 Mean (SD)	Data Interpretation out of 5 Mean (SD)
Rheumatology	3	20.91(0.82)	12.03(1.13)	18.15(2.10)	24.74(1.73)	3.95(0.65)
	4	21.08(0.98)	10.35(1.62)	17.5(1.85)	25.19(1.35)	3.46(0.80)
P-Value		0.598	0.031	0.419	0.165	0.002
Pediatric Surgery	3	21.08(1.47)	11.23(1.30)	18.36(2.16)	23.24(2.05)	4.34(0.46)
	4	22.18(1.35)	10.15(1.25)	18.39(1.74)	19.46(2.31)	4.04(0.71)
P-Value		0.696	0.494	0.240	0.663	0.501
Family Medicine	3	20.82(1.40)	11.96(1.23)	19.38(1.65)	24.08(1.96)	4.2(0.59)
	4	21.03(1.75)	11.34(1.14)	19.98(1.51)	24.3(1.32)	3.83(0.56)
P-Value		0.044	0.278	0.573	0.041	0.290
General Gyn/Obs	3	23.82(0.66)	11.37(1.79)	20.99(1.47)	24.08(1.96)	3.23(0.70)
	4	22.48(1.92)	10.55(1.25)	16.97(2.10)	25.28(1.51)	3.92(0.76)
P-Value		0.007	0.242	0.148	0.320	0.016
General Pediatric	3	23.0(0.94)	10.82(1.51)	18.71(1.90)	26.18(1.70)	4.23(0.53)
	4	22.8(2.21)	11.15(1.61)	18.09(2.17)	24.8(1.37)	3.65(0.66)
P-Value		0.003	0.805	0.390	0.092	0.125

The students’ Score in OSCE had a significantly strong correlation with quality of station monitors (r = 0.73, p=0.04), comprehensive coverage of content in OSCE (r = 0.721, p= 0.04) and flow between stations (r = 0.703, p=0.05). (Table-III)

**Table III:** Correlation of OSCE Score with the Students Perception on the Quality of OSCE.

Variable		Comprehensive coverage of content in OSCE	Quality of Station Monitors	Level of Difficulty	Flow Between Stations
Class Mean OSCE/ 30	r	0.721	0.729	-0.413	0.703
	p-value	0.044	0.040	0.310	0.052

Students gave a significantly high rating for female block coordinators (Mean 4.48 ± 0.11) compared to males (Mean 4.04 ± 0.22) for conducting PBL sessions (p=0.018). Female block coordinators also got significantly high rating (Mean 4.64 ± 0.16, p<0.007) for block organizations compared to Males (Mean 3.81 ± 0.41). There were no other significant differences among male and female block coordinators for conduction of block and other variables on the faculty evaluation form.

There was a weak to moderate and significant correlation between student’s score in OSCE with block organization (p<0.001), availability of clinical cases (p<0.003), organization of clinical activities (p<0.001), and commitment and motivation of faculty (p<0.001). Students’ perception of the coordinator’s Intelligent critical thinking was not related to students success (Table-III). Students perception of the commitment and motivation of the coordinator was also significantly correlated with their mean portfolio scores (p<.01). (Table-IV) Students perception of faculty’s commitment and motivation was strongly and significantly correlated with block organization (r=0.894, p<0.0001), availability of clinical cases (r=0.825, p<0.0001), performance of block coordinator (r=0.681, p<0.0001), cooperation with students (r=0.433, p<0.0001), helpfulness of the block coordinator (r=0.714, p<0.0001), and organization of clinical activities (r=0.818, p<0.0001).

**Table IV:** Correlation^1^ of students assessment score with Block Coordinator’s attributes perceived by the students.

Attributes of block coordinator		Cohort’s Mean Portfolio Out of 25	Cohort’s Mean Mid-term Out of 15	Cohort’s Mean Final written Out of 25	Cohort’s Mean OSCE Out of 30	Cohort’s Mean of Total Score
Group Rapport, Cooperation	r	-0.007	-0.031	0.029	0.079	0.027
	p-value	0.910	0.640	0.661	0.230	0.685
Intelligent Critical thinking of block coordinator	R	0.046	-0.085	-0.053	0.066	-0.017
	p-value	0.484	0.197	0.421	0.321	0.795
Balance of Participation by block coordinator	R	0.096	-0.059	-0.003	0.082	0.028
	p-value	0.148	0.369	0.967	0.213	0.675
Block Organization	R	0.143	0.031	0.028	.0215	0.124
	p-value	0.030	0.644	0.677	0.001	0.061
Clinical Cases made availabel by block coordinator	R	0.139	-0.055	0.049	0.198	0.126
	p-value	0.036	0.410	0.460	0.003	0.056
Organization of Clinical Activities within the Block	R	0.112	-0.003	0.023	0.246	0.136
	p-value	0.091	0.959	0.730	<0.0001	0.040
Commitment and motivation of block coordinator	R	0.162	-0.004	0.033	0.243	0.150
	p-value	0.015	0.956	0.621	<0.000	0.023

1 Spearman’s Rho (correlation Coefficient)

## DISCUSSION

The key findings of this study were that coordinator’s block organization capabilities were related to students success. Coordinator’s commitment and motivation was strongly correlated with block organization abilities, which in our opinion was the key factor that was also significantly related to student’s success. Interestingly students group that was with the Coordinator that had a postgraduate degree in medical education got high mean score only in midterm exam and portfolio, probably because she emphasized the importance of portfolio and gave better direction to student on writing their portfolios. Surprisingly student’s perception of the teacher’s intelligence was not related to their success, which is not in congruence with the Gathright et al. results.^3^

Our study found that student’s perceptions of Coordinator’s block organization skills, availability and commitment was related to availability of clinical cases and better organization of clinical activities. This result is in congruence with Gerbase et al. study where they found that Clerkship quality was related to better organization.^10^ It has been reported before that curricular design, administrative skills of the supervisors, learning resources and environment are significantly related to students success.^5^ We also saw that good organization of block availability of clinical cases was related to student success at the end of rotation which is a proxy indicator for quality of training in these clinical rotations. Our results are also similar to the Stern et al. study where they found significant correlations of USMLE scores with students ratings of the teachers.^11^

The single most significant factor in concordance with the Lieff et al. study was commitment and motivation of the faculty related to students’ success and Coordinator’s organization abilities.^6^ McLeod et al. and other authors had also identified motivation as one of the pedagogic concept that can help clinical teachers to understand their work and lead to better quality of teaching.^8^,^12^,^13^ Although this study did not use a standard tool to identify the motivation and commitment of faculty nevertheless our results do suggest that students perception of teachers motivation was significantly related to their block organization (inclusive of clinical activity organization and making better availability of clinical cases) capabilities which in turn was related to their success. Henceforth just as students can be motivated by their teachers; teachers can also be supported through faculty development programs (inclusive of competence development), that support the feeling of autonomy and aid in building intrinsic motivation.^8^,^13^ In our opinion motivation to teach should be a core component for faculty development programs.

### Limitations

The data is from one institution and the numbers are small for making generalization for the entire country. The student’s scores were only available as cohort’s mean score for each block and we could not do direct correlations for students exam scores with their perception of the block coordinator.

## CONCLUSIONS

Our study showed that faculty commitment and motivation was the most important factor that was (indirectly and directly) related to student’s success and faculty ratings by the students. The faculty training programs should focus on improving the block organization and management competencies particularly in Saudi Arabia and the Middle East for better student outcomes. We recommend that faculty development programs should include components identified through self-determination theory for improving the intrinsic motivation of faculty for better quality of education.^9^, ^14^ We also recommend further studies on scoring the motivation, self regulation and competence of faculty.
